# Ambient air pollution, smog episodes and mortality in Jinan, China

**DOI:** 10.1038/s41598-017-11338-2

**Published:** 2017-09-11

**Authors:** Jun Zhang, Yao Liu, Liang-liang Cui, Shou-qin Liu, Xi-xiang Yin, Huai-chen Li

**Affiliations:** 1 Jinan Municipal Center for Disease Control and Prevention, Jinan, Shandong 250021 China; 2 Department of Respiratory Medicine, Shandong Provincial Hospital Affiliated to Shandong University, Jinan, Shandong 250021 China; 3 Jinan Research Academy of Environmental Sciences, Jinan, Shandong 250000 China

## Abstract

We aimed to assess the acute effects of ambient air pollution and weather conditions on mortality in the context of Chinese smog episodes. A total of 209,321 deaths were recorded in Jinan, a large city in eastern China, during 2011–15. The mean concentrations of daily particulate matter ≤10 μm (PM_10_), fine particulate matter (PM_2.5_), sulfur dioxide (SO_2_) and nitrogen dioxide (NO_2_) were 169 μg/m^3^, 100 μg/m^3^, 77 μg/m^3^, and 54 μg/m^3^, respectively. Increases of 10 μg/m^3^ in PM_10_, PM_2.5_, SO_2_ and NO_2_ were associated with 1.11% (95% CI 0.96–1.26%), 0.71% (95% CI 0.60–0.82%), 1.69% (95% CI 1.56–1.83%), and 3.12% (95% CI 2.72–3.53%) increases in daily non-accidental mortality rates, respectively. Moreover, the risk estimates for these 4 pollutants were higher in association with respiratory and cardiovascular mortality. The effects of all the evaluated pollutants on mortality were greater in winter than in summer. Smog episodes were associated with a 5.87% (95% CI 0.16–11.58%) increase in the rate of overall mortality. This study highlights the effect of exposure to air pollution on the rate of mortality in China.

## Introduction

At present, air pollution is undoubtedly one of China’s most pressing public health issues. Northern China has experienced frequent and severe smog episodes since 2011, particularly in winter^1^. High concentrations of fine particulate matter (PM_2.5_) may not only cause substantially reduced visibility by scattering and absorbing sunlight but may also have detrimental effects on human health^2^. These severe smog episodes have been cause for unprecedented public concern regarding the health impacts of air pollution. To address this issue, the Chinese State Council released the “Atmospheric Pollution Prevention and Control Action Plan” on September 10, 2013 with the aim of reducing PM levels. Although many other industrialized countries have experienced severe air pollution problems in the past, including Belgium (Meuse Valley) in 1930^3^, the United States (Pennsylvania) in 1948^4^, and the United Kingdom (London) in 1952^5^, the intensity and geographic extent of and number of people affected by the recent serious air pollution events in China are unprecedented.

The smog that has been observed in China is a complex mixture of air pollutants that are present at high concentrations in a city or a region. This mixture includes such major components as particulate matter (PM; e.g., PM_10_ and PM_2.5_) and gaseous pollutants (e.g., nitrogen dioxide (NO_2_), carbon monoxide (CO), and sulfur dioxide (SO_2_))^1, 6, 7^. Many studies conducted worldwide have shown that short-term exposure to air pollution is associated with increased mortality and morbidity, especially due to cardiovascular and respiratory diseases; exacerbation of chronic respiratory conditions; and decreased lung function^6–9^. Although there existed a strong and useful body of evidence derived from research conducted in other countries, the exposure–response functions of other countries cannot simply be applied to the Chinese context because of differences in particulate composition and population characteristics. For example, in addition to the substantially higher PM_2.5_ concentrations in Chinese smog, the concentrations of SO_2_ and NO_2_ are now quite high as well^1^. Moreover, most Chinese studies on air pollution and mortality were conducted before 2010 and lacked PM_2.5_ data. Indeed, the consequences of China’s recent severe air pollution and smog episodes remain largely unknown.

The objective of this paper was to examine the acute effect of PM and gaseous pollutants on daily mortality rates in Jinan, a large urban city in eastern China. Daily ambient air pollutant, weather condition, and mortality data were systematically collected from 2011 to 2015. Obtaining such a large and up-to-date dataset allowed us to assess the effects of ambient air pollutants and weather conditions on mortality in the context of Chinese smog episodes.

## Results

Table 1 presents a summary of the daily air pollution concentrations and meteorological conditions in Jinan. The mean concentrations of PM_10_, PM_2.5_, SO_2_, and NO_2_ were 169 μg/m^3^, 100 μg/m^3^, 77 μg/m^3^, and 54 μg/m^3^, respectively

**Table 1:** Daily air pollutant levels and meteorological conditions in Jinan, China, 2011–2015.

	Minimum	25^th^ percentile	Median	75th percentile	Maximum	Mean	Standard deviation
PM_2.5_ (μg/m^3^)	16	60	86	122	443	100	60
PM_10_ (μg/m^3^)	32	115	152	203	693	169	82
SO_2_ (μg/m^3^)	12	40	58	97	429	77	56
NO_2_ (μg/m^3^)	13	38	50	65	165	54	22
Mean temperature (°C)	−9.4	5.0	16.5	24.0	34.0	14.8	10.6
Relative humidity (%)	13	40	55	70	100	56	20
Air pressure (Pa)	976	989	997	1004	1022	997	9
Wind speed (m/s)	0.2	1.8	2.3	3.1	8.4	2.5	1.1

The daily average counts of non-accidental, cardiovascular, and respiratory deaths are shown in Table 2. Data were obtained for the total of 209,321 deaths that were reported during the 5-year period between January 2011 and December 2015. During the study period, an average of 115 non-accidental deaths occurred each day, of which 65, 40 and 18 deaths were due to cardiovascular diseases, cerebrovascular and respiratory diseases, respectively. The daily mortality rate was higher for men than women and higher for people less than 65 years old than for those who were older than 65 years. The daily mortality rate exhibited a seasonal trend and was higher in the winter months than the summer months. The highest daily mortality rates were observed during smog episodes.

**Table 2:** Daily death counts by sex, age, season and cause of death in Jinan, China, 2011–2015.

	Minimum	25th percentile	Median	75th percentile	Maximum	Mean	Standard deviation
Daily death counts (No. of deaths)							
Total	12	99	111	127	220	115	23
Gender							
Men	10	56	63	72	124	65	12.8
Women	2	41	48	57	105	50	12.4
Age							
≤65 years	5	28	33	38	61	34	7.35
>65 years	6	67	76	91	166	80	19
Season							
Winter (451 days)	12	121	138	154	220	138	25
Summer (490 days)	69	91	100	109	158	101	15
Smog episode*							
Yes (269 days)	12	122	144	160	220	148	27
No (1557 days)	69	97	108	120	194	110	18
Cause of death							
Respiratory	1	14	17	22	43	18	6.3
Cardiovascular	6	52	61	75	149	65	18.4
Cerebrovascular	4	34	39	45	74	40	8.2

*Any period during which daily PM_2.5_ concentrations exceed 100 μg/m^3^ for three or more consecutive days was defined as a smog episode.

Correlations were identified between different air pollutants and meteorological parameters, and the 5 pollutants were significantly correlated with each other (Table 3). Table 3 shows the mean values of the Spearman coefficients for the correlations between air pollutants and weather conditions in Jinan. Positive correlations were identified between PM_10_ PM_2.5_, SO_2_, and NO_2_. Temperature was negatively correlated with SO_2_ and PM_10_.

**Table 3:** Spearman’s correlation between air pollutants and weather conditions in Jinan, China, 2011–2015.

	SO_2_	NO_2_	PM_10_	PM_2.5_	Temperature	Humidity	Pressure	Wind speed
SO_2_		0.752**	0.529**	0.360**	−0.207**	−0.223**	0.325**	−0.031
NO_2_	0.728**		0.533**	0.428**	−0.389**	−0.042	0.528**	−0.376**
PM_10_	0.563**	0.738**		0.800**	0.064	−0.260	0.110**	−0.038
PM_2.5_	0.649**	0.732**	0.854**		0.091	0.010	0.033	−0.184
Temperature	−0.479**	−0.254**	0.000	−0.190**		−0.185**	−0.711**	0.121**
Humidity	0.073**	0.222**	0.211**	0.416**	−0.096*		−0.040	−0.395**
Pressure	0.314**	0.246**	−0.084**	0.072*	−0.734**	−0.190		−0.200**
Wind speed	0.291**	0.509**	−0.213**	−0.329**	0.267**	−0.276**	−0.336**	

*P < 0.05. **P < 0.001. The summer months (May–September) are shown above the diagonal; the winter months (October–April) are shown below the diagonal.

**Figure 1:**
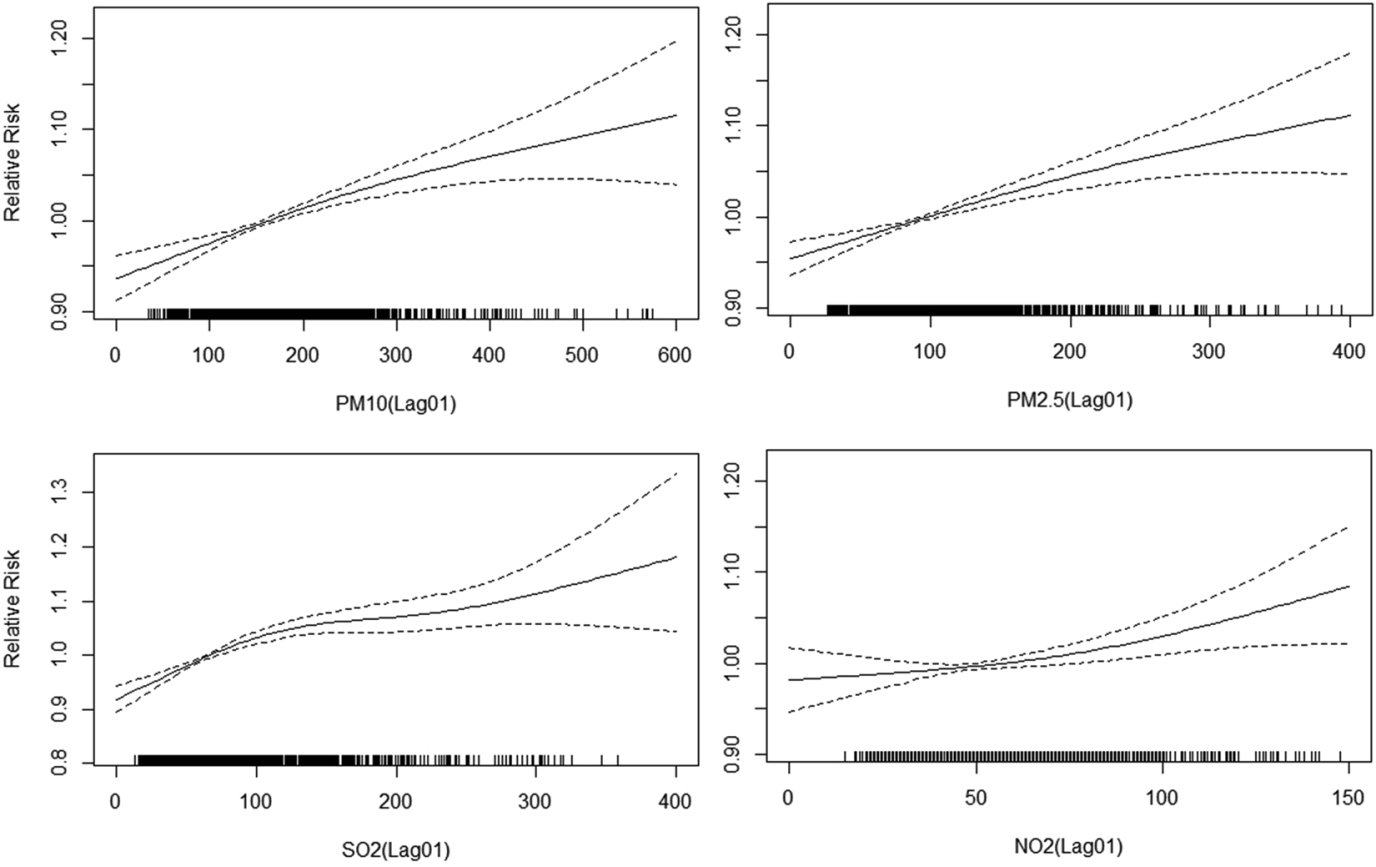
Associations between air pollutants (lag 0–1 day) and daily mortality in Jinan, China, 2011–2015. A natural cubic spline with 3 degrees of freedom for air pollutants was included in the single pollutant models, while controlling for day of the week, temperature, relative humidity, wind speed, and air pressure.

Table 4 shows the mean percent increases in daily mortality identified in association with air pollutants in Jinan during 2011–2015 using single- and multi-pollutant models. In the single-pollutant models for PM (PM_10_ and PM_2.5_), an increase of 10 μg/m^3^ of PM_10_ was associated with an increase of 1.11% (95% CI 0.96–1.26%) in the rate of non-accidental mortality, 1.40% (95% CI 1.18–1.61%) in the rate of cardiovascular mortality, 1.58% (95% CI 1.32–1.85%) in the rate of respiratory mortality, and 0.61% (95% CI 0.32–0.90%) in the rate of cerebrovascular mortality; additionally, an increase of 10 μg/m^3^ in PM_2.5_ was associated with an increase of 0.71% (95% CI 0.60–0.82%) in the rate of non-accidental mortality, 1.14% (95% CI 0.98–1.30%) in the rate of cardiovascular mortality, 1.02% (95% CI 0.82–1.21%) in the rate of respiratory mortality, and 0.43% (95% CI 0.30–0.55%) in the rate of cerebrovascular mortality. In the two/three-pollutant models, the associations between air pollutants and mortality were attenuated but remained statistically significant. The estimates for the effects of PM_2.5_ and PM_10_ on the rates of cardiovascular and respiratory mortality were higher than the estimates for the effects of these pollutants on the rate of non-accidental mortality. The gaseous pollutants (SO_2_ and NO_2_) were also significantly associated with non-accidental, cardiovascular, and respiratory mortality rates. The air pollutant and daily mortality associations tended to be linear (Fig. 1).

**Table 4:** Percent increase in the rate of overall non-accidental mortality and cause-specific mortality associated with a 10 μg/m3 increase in air pollutants at lag 0–1 day using single- and multi-pollutant models.

Pollutant‡ and model	Overall mortality (%)	Cardiovascular mortality	Respiratory mortality	Cerebrovascular mortality
PM_10_				
Single-pollutant model	1.11 (0.96–1.26)*	1.40 (1.18–1.61)*	1.58 (1.32–1.85)*	0.61 (0.32–0.90)*
+SO_2_	0.32 (0.14–0.49)*	0.38 (0.17–0.54)*	0.32 (0.01–0.62)*	0.14 (−0.07–0.34)
+NO_2_	0.78 (0.58–0.97)*	0.68 (0.39–0.96)*	1.07 (0.72–1.42)*	0.32 (0.10–0.54)*
+SO_2_ + NO_2_	0.48 (0.30–0.67)*	1.29 (0.88–1.70)*	0.58 (0.27–0.89)*	0.16 (−0.06–0.39)
PM_2.5_				
Single-pollutant model	0.71 (0.60–0.82)*	1.14 (0.98–1.30)*	1.02 (0.82–1.21)*	0.43 (0.30–0.55)*
+SO_2_	0.071 (−0.06–0.20)	0.33 (0.15–0.51)*	0.27 (0.11–0.43)*	0.065 (−0.08–0.22)
+NO_2_	0.36 (0.20–0.51)*	0.67 (0.45–0.89)*	0.52 (0.24–0.79)*	0.18 (0.01–0.35)*
+SO_2_ + NO_2_	0.22 (0.075–0.36)*	0.49 (0.29–0.70)*	0.33 (0.07–0.58)*	0.09 (−0.08–0.26)
SO_2_				
Single-pollutant model	1.69 (1.56–1.83)*	2.29 (2.09–2.48)*	2.52 (2.28–2.72)*	0.99 (0.82–1.15)*
+NO_2_	2.02 (1.82–2.23)*	2.44 (2.14–2.74)*	3.20 (2.83–3.56)*	1.07 (0.82–1.33)*
+PM_2.5_	1.76 (1.58–1.95)*	2.53 (2.26–2.79)*	2.78 (2.46–3.10)*	1.00 (0.78–1.22)*
+PM_10_	1.84 (1.66–2.01)*	2.28 (2.03–2.54)*	2.83 (2.53–3.14)*	1.00 (0.79–1.21)*
+NO_2_ + PM_2.5_	1.94 (1.75–2.15)*	2.47 (2.20–2.77)*	3.06 (2.87–3.27)*	1.05 (0.80–1.38)*
+NO_2 + _PM_10_	1.92 (1.73–2.13)*	2.50 (2.22–2.82)*	3.11 (2.70–3.58)*	1.03 (0.79–1.34)*
NO_2_				
Single-pollutant model	3.12 (2.72–3.53)*	4.80 (4.23–5.37)*	4.48 (3.77–5.19)*	1.83 (1.37–2.28)*
+SO_2_	−0.07 (−0.63–0.48)	0.88 (0.076–1.67)*	−0.57 (−1.57–0.43)	0.01 (−0.65–0.67)
+PM_10_	2.95 (2.37–3.53)*	4.34 (3.52–5.16)*	4.35 (3.32–5.37)*	1.48 (0.84–2.12)*
+PM_2.5_	2.40 (1.83–2.96)*	4.86 (4.06–5.66)*	3.70 (2.70–4.69)*	1.33 (0.71–1.96)*
+SO_2_ + PM_2.5_	0.17 (0.05–0.55)*	1.23 (0.41–3.69)*	1.07 (0.92–1.24)*	0.24 (−0.80–0.40)
+SO_2_ + PM_10_	0.38 (−0.49–0.27)	1.04 (0.33–3.28)*	−0.05(−0.59–0.48)	−0.14 (−0.90–0.62)

*P < 0.05. The analyses were adjusted for seasonality, day of the week, temperature, relative humidity, air pressure and wind speed.

**Figure 2:**
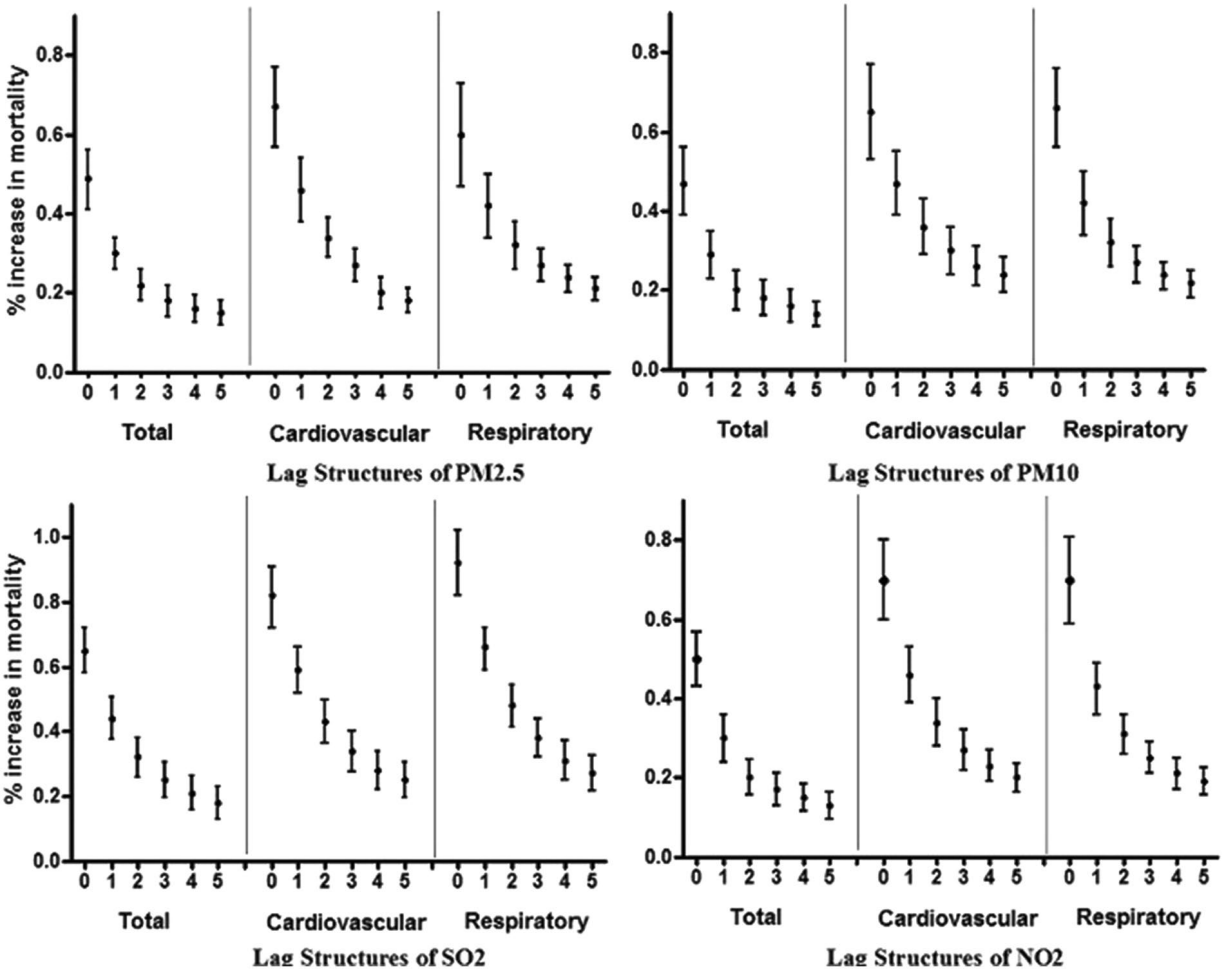
Percent increases in daily mortality associated with a 10 µg/m^3^ increase in PM_2.5_, PM_10_, SO_2_ and NO_2_ concentrations using different lag structures in Jinan, China, 2011–2015.

PM_2.5_, PM_10_ and NO_2_ demonstrated similar lag patterns in association with total non-accidental, cardiovascular and respiratory mortality (Fig. 2). For single-day lags, the rates of overall and cardiopulmonary mortality decreased from lag day 0 to 5 but remained statistically significant at lag day 5. The estimates for the effects of PM_2.5_, PM_10_ and NO_2_ on cardiovascular mortality were higher than those identified than the estimates for the effects of these pollutants on respiratory and overall non-accidental mortality. The effect of SO_2_ on respiratory mortality was higher than the effects of this pollutant on overall and cardiovascular mortality.

**Figure 3:**
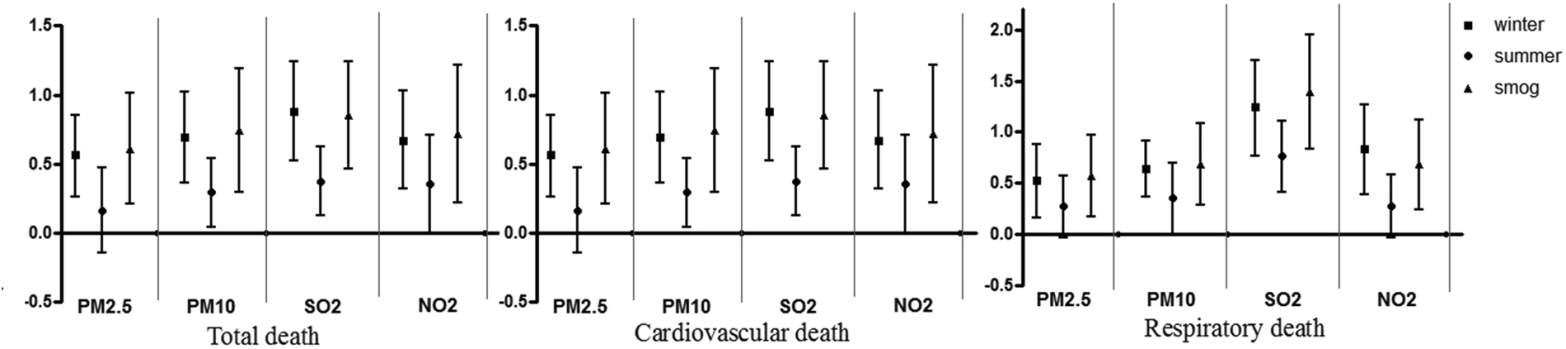
Estimated increases (and 95% confidence intervals) in daily mortality rates (overall non-accidental, respiratory, and cardiovascular) corresponding to a 10 μg/m^3^ increase in each pollutant during summer (May–September), winter (October–April) and smog periods (a total of 269 days) based on multi-pollutant models (lag 0–1 day).

Figure 3 presents the pooled effect estimates (mean and 95% CI) for the increases in overall non-accidental, cardiovascular and respiratory mortality associated with air pollutants in winter, summer and smog episodes using moving average lag models (lag 0–1). The associations between air pollutants and mortality differed by season. We observed that the associations between air pollutants and mortality were stronger during winter than during summer. Furthermore, we observed the greater estimates for the effects of the evaluated pollutants on overall, cardiovascular, and respiratory mortality during smog episodes.

In Table 5, we present the results of our investigation of the heterogeneous effects of smog episodes across different age and gender groups. A smog episode is associated with 5.87%, 6.31% and 7.10% increase in total non-accidental, cardiovascular, and respiratory mortality respectively. The estimates for the effects of smog episodes on mortality were higher in the older age groups (age 60–70 y and age > 70 y) than the younger age group (age < 60 y). The air pollutants demonstrate greater impacts on respiratory and cardiovascular mortality than overall non-accidental mortality across the different age and gender groups.

**Table 5:** Age- and gender-stratified effects of smog on mortality rates.

Pollutant^‡^ and model	Overall morality	Cardiovascular mortality	Respiratory morality
	% (95% CI)	% (95% CI)	% (95% CI)
Overall	5.87 (0.16–11.58)	6.31 (0.22–12.4)	7.10 (0.11–14.09)
Age	% (95% CI)*	% (95% CI)	% (95% CI)*
<60y	3.64 (−0.07–7.35)	4.02 (0.03–8.01)	4.41 (0.17–8.65)
60–70y	5.35 (−0.44–11.14)	4.91 (0.11–9.70)	5.71 (0.76–10.66)
>70y	8.32 (0.23–16.41)	8.86 (0.55–17.17)	9.47(0.23–18.71)
Gender	% (95% CI)	% (95% CI)	% (95% CI)
Male	4.97 (0.23–9.71)	6.02 (0.13–11.91)	6.88 (0.32–14.09)
Female	6.46 (−0.47–13.39)	6.70 (0.05–13.35)	7.33 (−0.89–15.56)

*P < 0.05.

## Discussion

We conducted an exploratory study of the short-term health effects of air pollution using data for the 209,321 non-accidental deaths that occurred during 2011–2015 in a highly polluted large urban city in eastern China. To our knowledge, this is the latest large study to systematically assess the acute effects of air pollution and smog episodes on mortality in China. We found that all the evaluated pollutants (PM_2.5_, PM_10_, SO_2_, and NO_2_) significantly impacted non-accidental mortality after adjustment for meteorological factors. Moreover, the risk estimates were greater in magnitude in association with respiratory and cardiovascular mortality than overall non-accidental mortality, and the effects of air pollutants on mortality rates were higher in winter than in summer. We also observed that smog episodes were typically positively and significantly associated with higher rates of mortality, and females and older persons were more vulnerable to smog exposure.

In the present analysis, an increase of a 10 μg/m^3^ in the 2-day moving average concentrations of PM_10_ corresponds to 1.11%, 1.40%, and 1.58% increases in the rates of total, cardiovascular, and respiratory mortality, respectively. Generally, the magnitudes of our effect estimates for PM_10_ were slightly higher than those reported in previous studies conducted in China and worldwide. For example, a study assessing data from 75 individual cities worldwide estimated that a 10 μg/m^3^ increase in PM_10_ corresponded to a 0.6% increase in the rate of overall mortality^10^. A USA study including data from 90 cities generated an estimate of 0.2% for PM_10_^11^, while a multicity study in four Asian cities (Bangkok, Seoul, Inchon, and Hong Kong) provided a pooled estimate of 0.55%^12^. The CAPES study, which included data registered in 17 Chinese cities (but not Jinan) between 1996 and 2008, showed that a 10 μg/m^3^ increase in PM_10_ was associated with a 0.35% increase in daily mortality rates^13^. An explanation for this difference might be that previous studies conducted in China and other developing counties used relatively old data. However, beginning in early 2011, China experienced unprecedented serious air pollution and severe smog events that were characterized by extremely high concentrations of ambient PM. In our study, we collected PM data from the most recent five years in Jinan, and the daily mean PM_10_ level identified in this study was much higher than those reported in previous studies in China. The stronger association observed between the level of particulate matter and the rate of mortality suggests that particulate air pollution may represent a major and growing public health problem in Chinese cities.

PM_2.5_ is a pollutant that is a major component of Chinese haze. However, real-time PM_2.5_ data have been only available since 2012 in most districts in China, after public outcry regarding the thick smog in China resulted in enhanced surveillance. While the body of epidemiological evidence regarding the association between short-term exposure to PM_10_ with health effects is substantial, relatively few studies have investigated fine particulate matter (measured as PM_2.5_) and evaluated its acute health effects. The results of a meta-analysis of studies published before 2011 showed that the pooled estimates for each 10 μg/m^3^ increase in PM_2.5_ were 1.04% (95% CI 0.52% to 1.56%) for the rate of non-accidental mortality, 1.51% (1.01 to 2.01%) for the rate of respiratory mortality, and 0.84% (0.41 to 1.28%) for the rate of cardiovascular mortality^14^. A previous study conducted in Beijing, China (2004–2008) reported that an increase of a 94 μg/m^3^ in PM_2.5_ was associated with a 1.3% (0.1 to 2.6%) increase in the rate of non-accidental mortality^15^. Our results are comparable with those of previous studies^14, 15^. For PM_2.5_, we found each 10 µg/m^3^ increase in this pollutant to be associated with a 0.71%, 1.14%, and 1.02% increase in the rates of total, cardiovascular, and respiratory mortality, respectively.

Our study confirmed previous findings suggesting that the associations between particulate matter and the rates of respiratory and cardiovascular diseases morality were stronger than the association between these pollutants and the rate of overall non-accidental mortality^16^. Moreover, our study showed that the associations between PM_2.5_ and PM_10_ and the rate of respiratory mortality risk were stronger than the associations between these pollutants and the rate of cardiovascular mortality^17, 18^. These results suggest that people with heart or lung diseases may be more sensitive to PM. Particulate matter has been reported to be associated with increased plasma viscosity^19^, changes in blood characteristics^20^, and indicators of anomalies in the autonomic function of the heart, including increased heart rate, decreased heart rate variability, and increased cardiac arrhythmias^21^. These findings provide possible pathways through which particulate matter may affect the cardiopulmonary system.

For SO_2_ and NO_2_, in the single-pollutant model, an increase of 10 μg/m^3^ was associated with increases of 1.69%, 2.29%, and 2.52% and 3.12%, 4.80%, and 4.48% in the rates of total, respiratory and cardiovascular mortality, respectively. Inclusion PM or NO_2_ in the model did not significantly influence the estimates for association between SO_2_ and mortality, while the estimates for association between NO_2_ and mortality decreased when SO_2_ was added. The associations between PM and the evaluated outcomes also became weaker after gaseous pollutants, particularly SO_2_, were included in the model. The estimates for the effect of SO_2_ on mortality in Jinan were slightly higher than those reported in most previous studies but were generally comparable with recent data from Chinese cities. A meta-analysis reported that the excess rate of non-accidental mortality associated with a 10 μg/m^3^ increase in SO_2_ was 1.00% (95% CI, 0.75 to 1.24%) in four Asian cities (Bangkok, Hong Kong, Shanghai and Wuhan)^22^. A recent time series analysis conducted in Ningbo (2009–2013) reported a 10 μg/m^3^ increase in SO_2_ to be associated with a 2.89% (95% CI 2.04–3.76%) increase in the rate of daily mortality^23^. However, some North American studies did not report a significant association between SO_2_ and mortality, which may be because of the very low levels of SO_2_ air pollution in these cities^24^. Our results suggested SO_2_ to have a greater impact on mortality in Jinan, and previous studies have suggested that the risk of mortality associated with exposure to SO_2_ may be greater in China than in Western countries.

We also investigated the effects of weather conditions on the association between air pollution and mortality, and we found that the adverse effects of air pollution were much stronger during the cool season in Jinan. One possible explanation may be that the composition of air pollution varies across seasons. From 2011 to 2015, the median levels of PM were all higher in the winter months than in the summer months. In general, atmospheric inversions are more likely to occur during the cold seasons, limiting the dispersion and dilution of pollutants emitted at the ground level. In addition, the combustion of fuel for space heating may results in increased PM_2.5_ emissions during the winter months. One could postulate that the ambient pollutant concentrations identified in this setting may reflect the mixed pollutants present in a smog episode, which may potentially have adverse health effects. Moreover, we also found that the smog episodes had a more substantial impact on mortality in older persons and females.

In conclusion, we identified significant associations between ambient air pollutant (PM_2.5_, PM_10_, SO_2_, and NO_2_) concentrations, smog episodes and daily mortality rates in Jinan, China. These estimates were robust due to the inclusion of meteorological factors and model specifications, and the pollutants exerted greater effects on the rates of respiratory and cardiovascular mortality than the rate of overall non-accidental mortality. In addition, the smog episodes had effects that were more detrimental on females and older persons. These findings highlight the importance and urgency of air pollution control; additionally, they call for the implementation of effective and evidence-based policies to improve air quality in China.

## Methods

### Mortality data

Jinan is a metropolitan area with a population of approximately 7 million located in the eastern part of China. Daily mortality data for Jinan during the 5-year period from January 2011 to December 2015 were obtained from the Jinan Municipal Center for Disease Control and Prevention, and the collected data included date of death, sex, and underlying cause of death according to the International Classification of Diseases, 10th Revision (ICD-10). A total of 209,321 deaths (117,857 male, 91,464 female) occurred during the five-year study period. The mortality data were categorized into deaths due to total non-accidental causes (ICD-10: A00-R99), cardiovascular disease (ICD-10: I00-I99), respiratory disease (ICD-10: J00-J99), and cerebrovascular disease (ICD-10: I60-I69). For overall mortality, the data were stratified by gender (female and male), and age (≤60y, 60–70y, and >70y).

### Pollution data

Mean hourly PM_10_, PM_2.5_, SO_2_, and NO_2_ concentration data were obtained from the Environmental Monitoring Center of Jinan for the same 5-year period for which mortality data were available. The data were obtained from 14 monitoring stations. The pollutant monitoring network spanned the entire region, including 12 sites located in the city and 2 sites located in a suburban county. We abstracted the daily 24-hr mean concentrations of PM_2.5_, PM_10_, SO_2_, and NO_2_. The daily air pollutant concentrations were calculated as the average of the concentrations recorded at the 14 stations.

### Meteorological data

We collected daily weather information for the period from January 1, 2011 to December 31, 2015 from the China Meteorological Science Data Sharing Service Network (http://cdc.cma.gov.cn/home.do). We downloaded the following data: daily mean temperature, air pressure, humidity, and wind speed.

### Statistical analysis

Generalized linear models (GLM) with quasi-Poisson regression were constructed using the MGCV package in R software to analyze the associations between daily concentrations of air pollutants and the number of all cause and cause-specific deaths. Considering the confounding effects of long-term trends, seasonal patterns and meteorological parameters, the smoothing spline functions involved in calendar time, daily mean temperature, relative humidity, wind speed and air pressure were applied. We used a natural cubic spline with 7 degrees of freedom per year for time. Based on Akaike’s information criterion (AIC), we specified the appropriate degrees of freedom in the smoothing spline functions for the evaluated weather conditions^25, 26^. The day of week was controlled for as a categorical variable. After establishing the basic model, we introduced the air pollutant concentrations into the models. The regression model can be written as follows:

$$\begin{array}{c}\mathrm{Log}\,E({\rm{Yt}})={\rm{intercept}}+{\rm{\beta }}\mathrm{Zt}+{\rm{ns}}({\rm{time}},{\rm{df}})+{\rm{DOW}}+{\rm{ns}}({\rm{temperature}},{\rm{df}})\\ \phantom{\rule{5em}{0ex}}+{\rm{ns}}({\rm{humidity}},{\rm{df}})+{\rm{ns}}({\rm{wind}}\,{\rm{speed}},{\rm{df}})+{\rm{ns}}({\rm{air}}\,{\rm{pressure}},{\rm{df}}),\end{array}$$

where E(Yt) represents the expected number of daily deaths on day t; ß represents the regression coefficient; Zt is indicative of the pollutant concentrations on day t; ns(time, df) denotes a smoothed function of calendar time with 7 df per year to control seasonality and longer-term trends; “DOW” is the dummy variable for day of the week; and ns(temperature/humidity/wind speed/air pressure, df) is a smoothed function of temperature, humidity, wind speed or air pressure with 3 df.

We examined these associations using different lag structures, including a single-day lag (from lag 0 to lag 5) and moving average lag (lag 0–1). For the single-day lag models, a lag of 0 days (lag 0) corresponded to the current-day air pollution concentration, and a lag of 1 day (lag 1) referred to the previous day’s pollutant concentrations. For the moving average lag models, lag 0–1 corresponded to a 2-day moving average of the air pollution concentrations recorded on the current and previous day. For each pollutant, we fitted both single-pollutant and multi-pollutant models to assess the stability of the associations. Stratified analyses were conducted according to sex, age and cause of death to identify populations who were potentially more sensitive to air pollution. To examine whether the effects of air pollutants on mortality differed by season in Jinan, we also evaluated the following time periods: summer months (June, July, August), winter months (January, February, and December) and smog episodes. The percentages increase in death associated with a 10 μg/m^3^ increase in each pollutant as well as their 95% confidence intervals (CI) were computed.

Any period during which daily PM_2.5_ concentrations exceed 100 μg/m^3^ for three or more consecutive days was defined as a smog episode^27^. We used the following models to compare the mortality differences between the smog episodes and the regular days.

$$\mathrm{Log}\,E({\rm{Yt}})={\rm{\alpha }}+{\rm{\beta }}\mathrm{smogt}\sum _{{\rm{i}}=1}^{{\rm{p}}}Si(Zit,\,df\,zi)$$

where yt is the number of deaths on day t; smogt is a dummy indicator for the smog episodes. ß represents the log-relative rate of mortality associated with a smoggy day. Zit are meteorological factors that are correlated with air pollution levels, and Si are their natural spline smooth functions.
